# A Molecular Genetic Linkage Map of *Eucommia ulmoides* and Quantitative Trait Loci (QTL) Analysis for Growth Traits

**DOI:** 10.3390/ijms15022053

**Published:** 2014-01-28

**Authors:** Yu Li, Dawei Wang, Zhouqi Li, Junkun Wei, Cangfu Jin, Minhao Liu

**Affiliations:** 1 College of Forestry, Northwest A&F University, Yangling 712100, Shaanxi, China; E-Mails: liyu@nwsuaf.edu.cn (Y.L.); daweiwon@163.com (D.W.); jun_kun@126.com (J.W.); jincangfu2008@nwsuaf.edu.cn (C.J.); liuminhao@nwsuaf.edu.cn (M.L.); 2 College of Forestry, Southwest Forestry University, Kunming 650224, Yunnan, China

**Keywords:** *Eucommia ulmoides*, molecular markers, genetic linkage map, growth traits, QTL

## Abstract

*Eucommia ulmoides* is an economically important tree species for both herbal medicine and organic chemical industry. Effort to breed varieties with improved yield and quality is limited by the lack of knowledge on the genetic basis of the traits. A genetic linkage map of *E. ulmoides* was constructed from a full-sib family using sequence-related amplified polymorphism, amplified fragment length polymorphism, inter-simple sequence repeat and simple sequence repeat markers. In total, 706 markers were mapped in 25 linkage groups covering 2133 cM. The genetic linkage map covered approximately 89% of the estimated *E. ulmoides* genome with an average of 3.1 cM between adjacent markers. The present genetic linkage map was used to identify quantitative trait loci (QTL) affecting growth-related traits. Eighteen QTLs were found to explain 12.4%–33.3% of the phenotypic variance. This genetic linkage map provides a tool for marker-assisted selection and for studies of genome in *E. ulmoides*.

## 1. Introduction

*Eucommia ulmoides* Oliver (2*n* = 34), the single extant species of the genus *Eucommia* (Eucommiaceae), is strictly a dioecious perennial tree [1]. It is an economically important plant for both herbal medicine and organic chemical industry. Chemical constituents (e.g., phenylpropanoids and flavonoids) in the bark and leaves have high pharmacological activities and health care functions of lowering blood pressure and blood sugar, resisting oxidation and mutation, improving the health, strengthening the body, promoting metabolism and relieving tiredness [2–5]. The whole plant except xylem contains Eucommia-rubber which is an important raw material in the chemical industry. Eucommia-rubber is a hard rubber with thermoplasticity, and it has properties that are similar to those of plastic [6]. Historically, only the bark was officially recognized as a traditional Chinese herbal drug. In recent years, the bark of *E. ulmoides* also was used to produce Eucommia-rubber in China, Russia and Japan. To improve the quality and yield of the bark, height and diameter growth were the main parameters for selection [7].

Conventional breeding of *E. ulmoides* has mainly focused on the selection of promising plants from existing natural populations. These selected plants were propagated vegetatively and released as clones. Recently, these cultivars were used as parents in crossbreeding. However, classical breeding often takes decades to fully evaluate and release new cultivars. The ability of *E. ulmoides* breeders to select promising parents for crossing, and to identify progenies with favorable combinations of characters, is hampered by the limited knowledge of the genetic basis of economically important traits. The speed and precision of breeding can be improved by the development of genetic linkage maps. Such genetic linkage maps can facilitate the development of diagnostic markers for polygenic traits and the identification of genes controlling complex phenotypes. The linked molecular markers identified in quantitative trait loci (QTL) analysis could then potentially be used in breeding practice via marker-assisted selection, where the selection is based on DNA sequence rather than the phenotype.

For forest trees, given the high genetic load and long generation time, segregating populations derived from crosses between inbred lines are not available. To circumvent this limitation, a pseudo-testcross approach is generally used to construct linkage maps from full-sib populations. Combined with the pseudo-testcross strategy, molecular markers such as random amplified polymorphic DNA (RAPD), sequence related amplified polymorphism (SRAP), amplified fragment length polymorphism (AFLP), inter-simple sequence repeat (ISSR) and simple sequence repeat (SSR) have been used extensively for the preparation of linkage maps of a number of tree species [8–12]. In a pseudo-testcross, only dominant markers that segregate in a 1:1 ratio are used to build separate molecular maps for each parent. Considering modern marker technologies are available for full-sib populations, markers that segregate in 3:1 (dominant), 1:2:1 (co-dominant) and 1:1:1:1 (co-dominant) ratios, in addition to 1:1, can be used to integrate individual linkage maps [13–15]. Using genetic linkage maps, QTL analysis have been conducted for traits of leaf, growth, vegetative propagation, wood quality, resistance, yield, flowering and fruiting in tree species [16–21].

In order to construct a genetic linkage map of *E. ulmoides*, we produced a F_1_ mapping population from the cross between a wild genotype Xiaoye and a cultivar Qinzhong No. 1. The female parent Xiaoye originated from the forest at Yantuo, Lingbao, Henan. The male parent Qinzhong No. 1 was one of the four earliest cultivars [22], and it was planted in the museum garden of Northwest A&F University, Yangling, Shaanxi. Xiaoye and Qinzhong No. 1 were chosen as parents because they differ in important quantitative traits. For instance, Xiaoye has late budding and flowering times, low content of secondary metabolite, small leaves, and smooth bark, whereas Qinzhong No. 1 has early budding and flowering times, high content of secondary metabolite, large leaves, and rough bark. Besides, Qinzhong No. 1 is an excellent cultivar, fast growing and with high resistance to drought and cold. In this study, we present a genetic linkage map of *E. ulmoides* based on SRAP, AFLP, ISSR and SSR markers. Results from our QTL analysis for height and basal diameter, measured over four consecutive years, are reported.

## 2. Results

### 2.1. Molecular Markers

The 2048 SRAP primer combinations (Table 1), 64 AFLP primer combinations (Table 2), 100 ISSR primers and 19 SSR primer combinations were tested on the parents and a small set of the DZ0901 progeny. Of these, 131 SRAP primer combinations, 18 AFLP primer combinations, 16 ISSR primers (Table 3) and 17 SSR primer combinations were more informative and were used for amplification (Table 4). Of the 1604 polymorphic SRAP markers (with an average of 12.2 polymorphic markers per primer combination), 305 were lmxll markers (1:1), 326 were nnxnp markers (1:1), 382 were hkxhk markers (3:1), 18 were hkxhk markers (1:2:1), 13 were egxef markers (1:1:1:1), eight were abxcd markers (1:1:1:1), and 552 (34.4%) showed segregation distortion (*p* < 0.05) (Table 4). Of the 295 polymorphic AFLP markers (with an average of 16.4 polymorphic markers per primer combination), 141 were lmxll markers (1:1), 108 were nnxnp markers (1:1), 22 were hkxhk markers (3:1), and 24 (8.1%) showed segregation distortion (*p* < 0.05) (Table 4). Of the 111 polymorphic ISSR markers (with an average of 6.9 polymorphic markers per primer), 27 were lmxll markers (1:1), 23 were nnxnp markers (1:1), 31 were hkxhk markers (3:1), and 30 (27.0%) showed segregation distortion (*p* < 0.05) (Table 4). Of the 132 polymorphic SSR markers (with an average of 7.8 polymorphic markers per primer combination), 33 were lmxll markers (1:1), 42 were nnxnp markers (1:1), 26 were hkxhk markers (3:1), six were hkxhk markers (1:2:1), seven were egxef markers (1:1:1:1), one were abxcd markers (1:1:1:1), and 17 (12.9%) showed segregation distortion (*p* < 0.05) (Table 4). In total, 2142 polymorphic markers were scored from 182 primer combinations or primers with an average of 11.8 polymorphic markers per primer combination or primers. Of these, 623 (29.0%) markers showed segregation distortion (*p* < 0.05) and were excluded from mapping. Only 1519 markers conforming to Mendelian segregation ratios were used for the construction of the genetic linkage map.

### 2.2. Genetic Linkage Map

**Figure 1.**
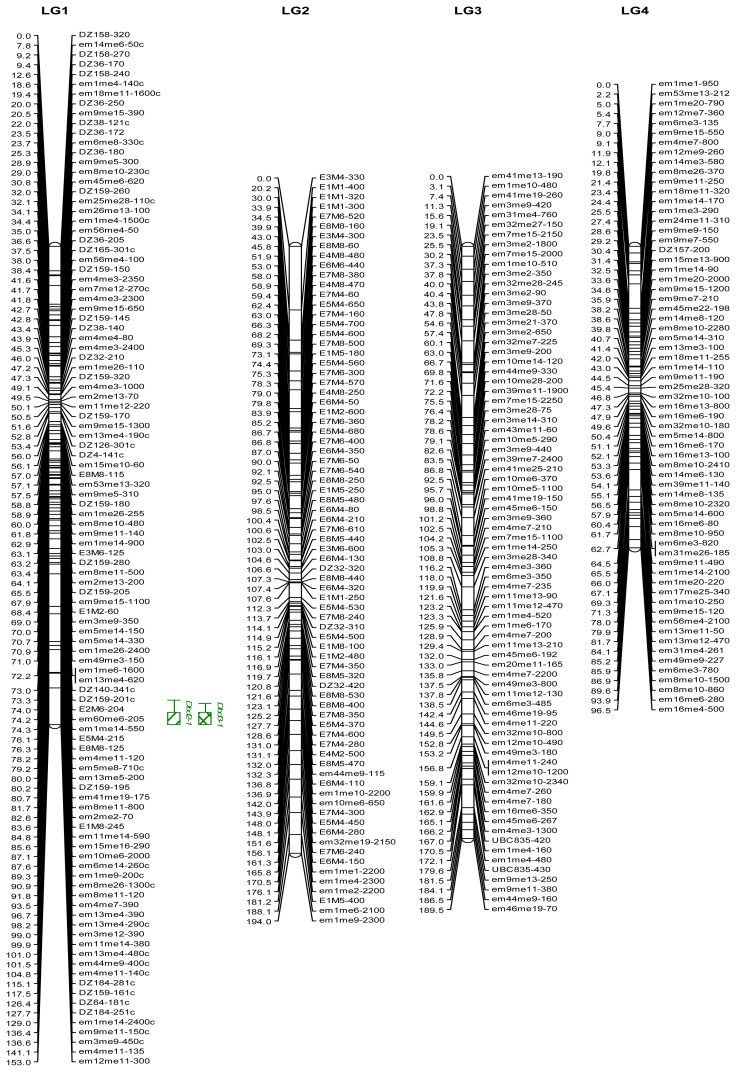

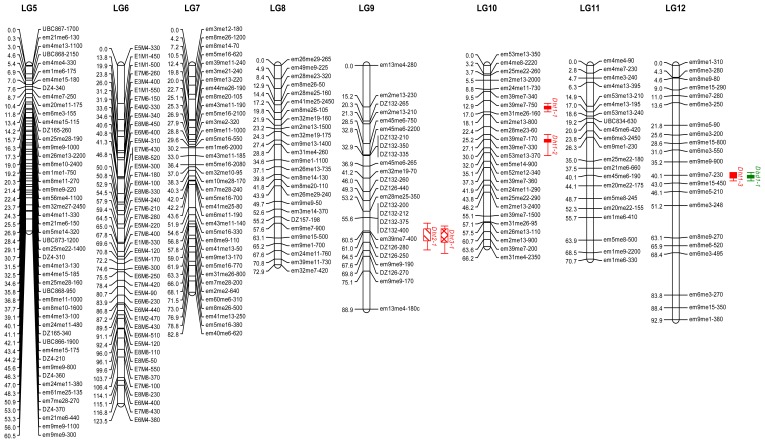

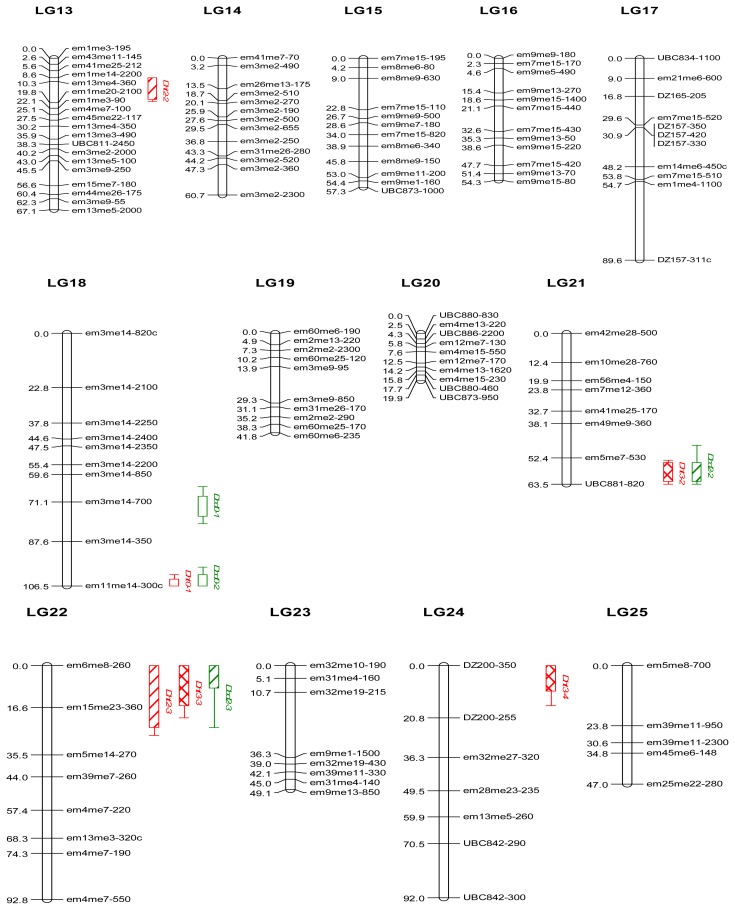
A genetic linkage map of *Eucommia ulmoides* based on DZ0901 population and quantitative trait loci (QTLs) for growth-related traits. Map units (cM) shown on the left of each linkage group (LG) were calculated by Kosambi mapping function. Markers are on the right side of the linkage groups. The markers are named with the code referring to the corresponding primer or primer combination (see Tables 1–3), followed by the estimated size of the DNA fragment in nucleotides. The map contains a total of 706 molecular markers, 515 SRAP markers, 117 AFLP markers, 18 ISSR markers and 56 SSR markers. The map spans 25 linkage groups (LG1–LG25) and covers a total genetic distance of 2133 cM. 1-LOD and 2-LOD support intervals of each QTL are marked by *thick* and *thi*n *bars*, respectively. Red bars represent QTLs for height. Green bars represent QTLs for basal diameter. Blank bars represent QTLs for the traits measured in 2010. Solid bars represent QTLs for the traits measured in 2011. Bars filled with one-sided hatch lines represent QTLs for the traits measured in 2012. Bars filled with two-sided hatch lines represent QTLs for the traits measured in 2013.

The genetic linkage map (DZ0901) consisted of 706 markers distributed over 25 linkage groups (LG) covering 2133 cM (Table 5 and Figure 1). The number of mapped makers per LG ranged from 5–106 with a mean of 28.2. The map size of the LGs ranged from 19.9–194.0 cM with a mean of 85.3 cM. The average map distance between adjacent markers was 3.1 cM. In addition, 165 markers distributed over 25 triplets and 45 doublets. There were 628 unlinked markers and 20 markers that successfully linked with a group but could not be ordered. Since our estimate of *E. ulmoides* genome length was 2403 cM, the genetic linkage map constructed in our study covered approximately 89% of the genome.

### 2.3. Growth Traits and QTL Analysis

**Figure 2.**
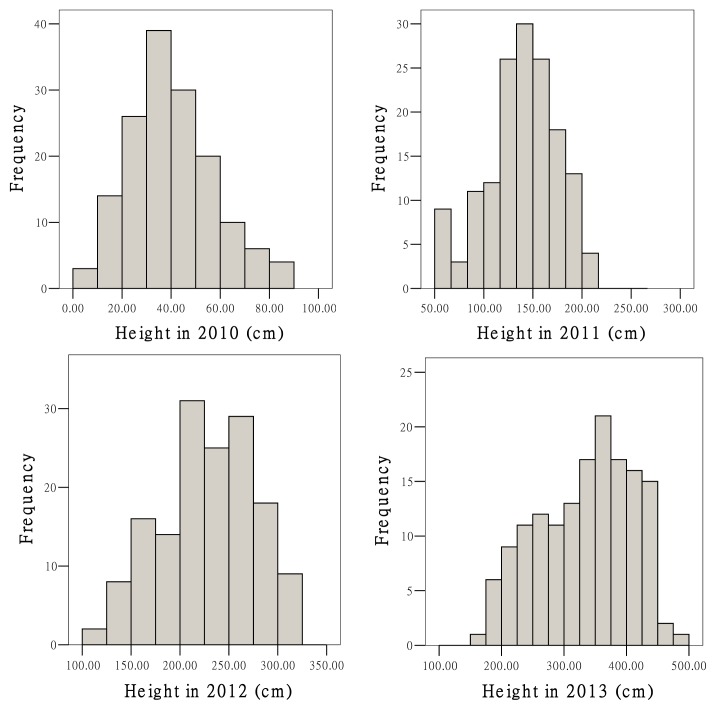

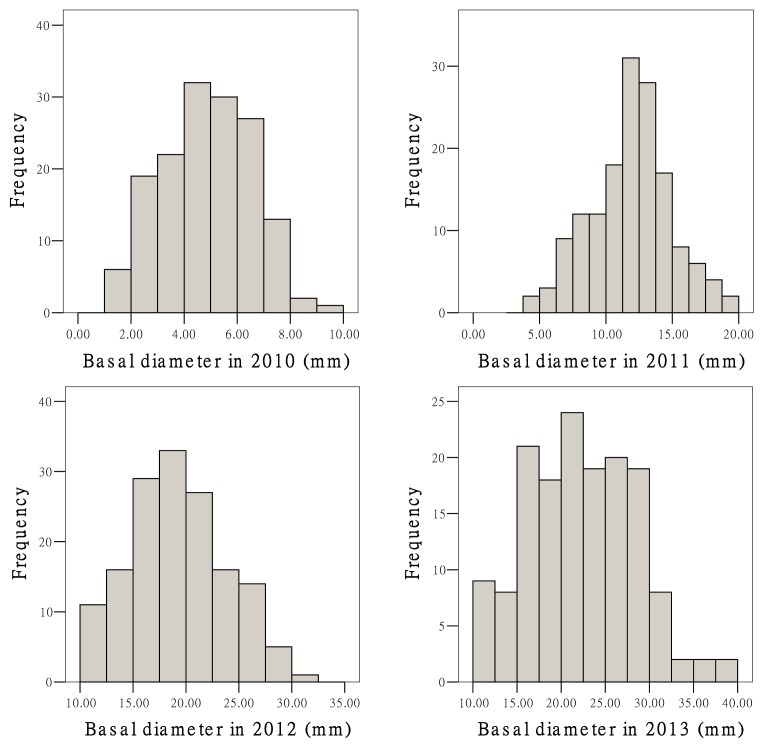
Frequency distributions of growth traits for DZ0901 population.

A high degree of genetic variation was found for height and basal diameter (Table 6). Figure 2 showed the frequency distributions of these traits. Pearson correlation analyses showed significant correlations between height and basal diameter and moderate weak correlations over years (Table 7).

The genetic linkage map of DZ0901 was used to search for putative QTLs (Table 8 and Figure 1). Eleven height QTLs were detected. In 2010, one height QTL was located on LG18 and explained 17.1% of the phenotypic variation. In 2011, three additional height QTLs were located on LG10, LG10 and LG12, and explained 29.7%, 27.7% and 22.8% of the phenotypic variation, respectively. In 2012, three height QTLs were located on LG9, LG13 and LG22, and explained 12.6%, 12.4% and 33.3% of the phenotypic variation, respectively. In 2013, two height QTLs identified at similar genomic regions as the height QTLs in 2012 were located on LG9 and LG22, and explained 13.5% and 25.3% of the phenotypic variation, respectively. Other two height QTLs in 2013 were located on LG21 and LG24, and explained 26.6% and 27.1% of the phenotypic variation, respectively. Four basal diameter QTLs were identified at similar genomic regions as the height QTLs. In 2010, one basal diameter QTLs was located on LG18 and explained 13.4% of the phenotypic variation. In 2011, one basal diameter QTLs was located on LG12 and explained 20.2% of the phenotypic variation. In 2012, two basal diameter QTLs were located on LG21 and LG22, and explained 25.1% and 21.4% of the phenotypic variation, respectively. Three additional basal diameter QTLs were detected on LG18, LG1 and LG1, and explained 29.8%, 17.7% and 16.8% of the phenotypic variation, respectively. Four of the 18 QTLs were significant, and they were *Dht0-1*, *Dht1-1*, *Dht1-2* and *Dbd0-2*. Other QTLs were not significant, but they had a LOD score greater than 3.0. Flanking markers and QTLs supported by Kruskal-Wallis nonparametric test were indicated in Table 8.

## 3. Discussion

### 3.1. Marker Amplification

SRAP has been recognized as an efficient and useful marker system [12,13,23]. It has several advantages such as simplicity, high throughput, numerous co-dominant markers and easy isolation of DNA fragments for sequencing, and it targets open reading frame regions. In this mapping population, SRAP analysis was an efficient method for generating polymorphic markers. Every primer combination gave at least six polymorphic markers with an average of 12.2 per primer combination. This is comparable to the polymorphism in other tree mapping projects using SRAP analysis [12,13].

It is known that AFLP marker produces a larger number of polymorphic fragments than other techniques. In our study, the average number of polymorphic DNA fragments per primer combination was 16.4. This is comparable to the average obtained in other tree mapping projects [9,17], but lower than the average reported for mapping using interspecific crosses [24,25]. As reported on many plant species [24,25], AFLP markers were dominant in this mapping population.

ISSR analysis has been used successfully to construct genetic linkage map of many tree species [9,10,12]. In these studies, ISSR markers were highly polymorphic and tended to be evenly distributed throughout genomes. Besides, the ISSR analysis was faster and easier than the AFLP analysis. However, it was less efficient with an average of 6.9 polymorphic markers per primer and had a limited number of primers. Like AFLP marker, ISSR markers were dominant in this mapping population.

SSR markers are typically co-dominant, highly polymorphic and highly reproducible across laboratories. They are also useful for comparing and combining linkage maps from different mapping populations. Furthermore, many SSR markers are transferable across related species [26,27]. Unfortunately, *E. ulmoides* is the single extant species of the genus *Eucommia*, and there are fewer available SSR primer combinations. In this study, we used 19 SSR primer combinations. Additional SSR markers are currently being added to better bridge this map with future *E. ulmoides* maps.

### 3.2. Segregation Distortion

In this study, 29% of the markers showed segregation distortion. We excluded these markers to obtain a more accurate genetic linkage map because distorted markers can affect the mapping accuracy by overestimating the map distances and causing marker clustering [9,28]. Also, the order of markers on linkage groups may be affected by segregation distortion [29]. We may have lost some information by excluding the distorted markers. However, we obtained a genetic linkage map covering approximately 89% of the estimated *E. ulmoides* genome with an average of 3.1 cM between adjacent markers. In a follow-up study, we intend to map these distorted markers using a larger mapping population and co-dominant markers.

Segregation distortion has been reported frequently in woody species. The percentage of markers showing segregation distortion was highly variable: 47% in spruce [28], 38% in pear [25], 29% in citrus [12], 18% in *Salix* [8], 9% in grape [9], 8.5% in *Populus* [13] and 1.8% in peach [30]. Compared to these data, distorted frequency in this study appeared to be intermediate (29%). Many biological mechanisms have been implicated in causing segregation distortion including divergence of the parental genotypes [13,25,31], chromosome loss [32], genome size differences [31], genetic load and recessive lethal alleles [33], meiotic drive locus [34], and gametic and zygotic selection [35,36]. In the present study, the female parent Xiaoye was a wild genotype from the forest in Henan province. The male parent Qinzhong No. 1 was a cultivar produced by controlled breeding, and it was planted in the museum garden of Northwest A&F University. They differ in traits of growth, phenology, morphology and content of secondary metabolite. Thus, the divergence of the parental genotypes may contribute to the observed segregation distortion.

### 3.3. Genetic Linkage Map

We constructed a genetic linkage map of *E. ulmoides* based on the segregation of SRAP, AFLP, ISSR and SSR markers as a first step towards understanding the *E. ulmoides* genome. The total map distance was 2133 cM, and the average map distance between adjacent markers was 3.1 cM. The present map covers a significant portion of the *E. ulmoides* genome, which should provide adequate coverage of the genome to begin QTL analysis. *E. ulmoides* is a diploid species with 2*n* = 34. The number of linkage groups was more than the number of haploid chromosomes of *E. ulmoides*. The presence of more than 17 linkage groups may be due to some gaps preventing connection between groups belonging to the same chromosome. However, gaps in the genetic linkage maps, resulting in two or more linkage groups per chromosome, are common in tree species even with large numbers of markers [8,11,13,15]. In future work, more co-dominant and functional markers are needed to be added to this genetic linkage map in order to fill the gaps, integrate some linkage groups and cover the entire genome.

### 3.4. QTL Analysis

It is often assumed that a quantitative trait exhibits continuous variation because of the interaction of environmental effects and multiple genes of small and cumulative effects. In the present study, we were able to detect QTLs with moderate to large effect for growth-related traits. The estimated magnitude of the individual QTL effect ranged from 12.4%–33.3% of the phenotypic variance. Our results agree with other QTL studies in tree species indicating that growth-related traits may in part be controlled by a few genes with large effect. In an F_2_ population based on an interspecific cross of *Populus*, Bradshaw and Stettler [37] reported that effects of single QTL for growth-related traits explained 24%–33% of the phenotypic variation. In an interspecific backcross family of white poplar, Zhang *et al*. [38] found that four QTLs for stem volume explained 35.8% of the total phenotypic variance. In a tetraploid hybrid F_2_ population of *Salix*, most of the QTL for the different growth-related traits each explained around 12% of the phenotypic variation, with a few exceptions explaining more than 20% of the variation [39]. Furthermore, in an intraspecific cross of *Salix*, 11 QTLs were identified for growth-related traits with each QTL explaining 14%–22% of the phenotypic variance [8].

Four basal diameter QTLs were identified at similar genomic regions as the height QTLs. This was not surprising because of the high correlation coefficient between height and basal diameter. The Pearson correlation coefficients were 0.80 in 2010, 0.83 in 2011, 0.66 in 2012 and 0.72 in 2013, respectively (Table 7). This suggested that height and basal diameter growth in *E. ulmoides* had common genetic components. The clustering of QTLs controlling highly correlative growth-related traits have been reported in other tree species of *Populus* [37,38], *Salix* [8,39], *Eucalyptus* [18,19,40] and apple [41].

No QTL was consistently expressed over the four years. However, QTL *Dht2-1*, *Dht2-3* and *Dbd2-1* in 2012 were identified at similar genomic regions as the QTL *Dht3-1*, *Dht3-3*, and *Dbd3-1* in 2013, respectively. A similar result of QTL analysis for height and basal diameter in radiata pine was reported by Emebiri *et al.* [42], who observed that none of the putative QTL positions detected at any one age was strongly expressed at all of the four stages of measurement and that 45% of putative QTLs significant at one age were also detected at a subsequent age. For growth-related traits, QTL instability has been reported frequently in tree species [8,15,37,40,41,43]. Verhaegen *et al.* [40] did not find the same QTLs over three consecutive years for growth-related traits in hybrid *Eucalyptus*. In rubber tree, QTLs detected during the summer were different from the QTLs detected during the winter for height and girth growth [15]. To explain this phenomenon, Verhaegen *et al.* [40] assumed that a set of regulatory genes may differentially control the temporal expression of the genes controlling a trait or that different sets of regulatory factors may be involved during different periods of time. Kenis and Keulemans [41] proposed that genetic control of these traits is largely influenced by environmental factors and probably changes as the tree matures.

To be able to utilize marker-assisted selection successfully in a breeding program, the molecular markers should be consistently found in various environments and show a large effect on the trait. In this study, we have considered only the first four years of a tree’s life, and the phenotypic assessment was undertaken in a single environment. Therefore, further QTL analysis under different environmental conditions over the years is necessary for providing additional insights on the pattern and stability of the growth QTLs.

## 4. Experimental Section

### 4.1. Plant Material

The population consisted of 152 F_1_ individuals that resulted from the cross between a wild genotype Xiaoye and a cultivar Qinzhong No. 1. Controlled pollination was carried out in the spring of 2009 at Yantuo, Lingbao, Henan, and seeds were collected in October and stored at 4 ºC. In March 2010, seeds were sown in a substrate with humus, sand and soil (1:1:1 mix) in plastic cups. Subsequently, seedlings were transplanted to the flat in a greenhouse when they had grown to a height of approximately 20 cm. The progenies were planted in the field in March 2011 at the nursery of Northwest A&F University, Yangling, Shaanxi. The F_1_ population was designated as “DZ0901”.

### 4.2. DNA Extraction

DNA was extracted from young leaves of the 152 F_1_ individuals and the two parental trees according to a modified CTAB procedure [44]. DNA quality was visually assessed on a 1% agarose gel by electrophoresis, and the concentration was determined using a NanoDrop ND-1000 spectrophotometer (NanoDrop Technologies Inc., Wilmington, DE, USA).

### 4.3. SRAP Analysis

SRAP analysis was performed according to Li and Quiros [23] with some modifications. Approximately 50 ng DNA was added to a mixture containing 2.5 mM MgCl_2_, 0.2 mM dNTPs, 0.4 mM of each primer, 1× PCR buffer and 1.5 U Taq DNA polymerase for a total volume of 25 μL. PCR parameters were as follows: 5 min at 94 ºC, 5 cycles of 94 ºC for 1 min, 35 ºC for 1 min and 72 ºC for 1.5 min, 30 cycles of 94 ºC for 1 min, 50 ºC for 1 min and 72 ºC for 1.5 min, and a final extension of 10 min at 72 ºC. DNA fragments were separated by electrophoresis on 8% non-denaturing polyacrylamide gel and visualized by silver staining. The SRAP primers used in this study are listed in Table 1.

### 4.4. AFLP Analysis

AFLP analysis consisting of genomic DNA digestion with *EcoR*I and *Mse*I restriction enzymes, adapter ligation, pre-amplification, and selective amplification using *EcoR*I plus three and *Mse*I plus three selective nucleotide primers were similar to those from Vos *et al.* [45] with modifications described by Wang *et al.* [46]. The following cycling parameters were used for pre-amplification: 94 ºC for 2 min, 30 cycles of 94 ºC for 30 s, 56 ºC for 30 s and 72 ºC for 80 s, and a final extension of 5 min at 72 ºC. PCR procedure for selective amplification was as follows: 94 ºC for 2 min, 14 cycles of 94 ºC for 30 s, 65 ºC for 30 s (reduced by 0.7 ºC/cycle) and 72 ºC for 80 s, 23 cycles of 94 ºC for 30 s, 56 ºC for 30 s and 72 ºC for 80 s, followed by 5 min at 72 ºC. DNA fragments were separated by electrophoresis on 6% denaturing polyacrylamide gel and visualized by silver staining. The AFLP primers used in this study are listed in Table 2.

### 4.5. ISSR Analysis

The protocols of Zietkiewicz *et al.* [47] for ISSR were adapted. Reaction mixture was as described above for SRAP except that a single primer was used. Thermal cycling conditions were as follows: 94 ºC for 4 min, followed by 38 cycles of 94 ºC for 30 s, 45 s at the locus-specific annealing temperature and 72 ºC for 1.5 min, and then a final extension step of 72 ºC for 5 min. PCR products were detected as described above for SRAP. The 100 primers were from the #9 ISSR primer kit (801–900) of the Biotechnology Laboratory, University of British Columbia (UBC, Vancouver, BC, Canada).

### 4.6. SSR Analysis

The SSR reaction mixture was as described above for SRAP. Thermal cycling conditions were described by Deng *et al.* [48]: 4 min at 94 ºC, locus-specific amplification cycles of 50 s at 94 ºC, 50 s at the locus-specific annealing temperature and 90 s at 72 ºC, and a final extension step for 10 min at 72 ºC. PCR products were detected as described above for AFLP. Nineteen SSR primer combinations developed for *E. ulmoides* by Deng *et al.* [48] were used in this study.

### 4.7. Segregation Analysis and Map Construction

Data of segregating markers was analyzed as a “cross-pollinated” population using JoinMap 4.0 [49]. Deviation from expected Mendelian ratio was determined using a chi-square test. The marker placement was determined using a minimum LOD threshold of 4.0 (Plant Research International B.V. and Kyazma B.V., Wageningen, Gelderland, The Netherlands, 2006), a recombination fraction threshold of 0.45, ripple value of 1.0 and jump threshold of 5.0, and mapping distances were calculated using the Kosambi (Plant Research International B.V. and Kyazma B.V., Wageningen, Gelderland, The Netherlands, 2006) mapping function. The genetic linkage map was plotted using MapChart 2.2 [50]. To estimate observed genome coverage, the expected genome length of each linkage group was calculated by multiplying the observed length by (m + 1)/(m − 1), where m is the number of markers in that linkage group, and the estimated genome length was the sum of revised length of all linkage groups [51]. Observed genome coverage was assessed by dividing the observed genome length by the estimated genome length.

### 4.8. Growth Traits Assessment and QTL Analysis

Height and basal diameter were measured to evaluate the growth of progenies in October from 2010–2013. The descriptive statistics, the skewness of the distributions and Pearson correlations of traits were calculated using SPSS 13.0 (SPSS Inc., Chicago, IL, USA, 2004) for Windows. QTL analysis was done using MapQTL 5.0 (Plant Research International B.V. and Kyazma B.V., Wageningen, Gelderland, The Netherlands, 2004) [52]. Kruskal-Wallis nonparametric test, interval mapping (IM) and multiple QTL mapping (MQM) were performed for each trait. In MQM, the markers closest to the QTL peaks detected by IM were used as cofactors. The limit of detection (LOD) thresholds was estimated with a 1000-permutation test. The QTLs with LOD values higher than the genome-wide threshold at *p* < 0.05 were considered significant. However, those QTLs with a LOD score greater than 3 and smaller than the threshold were also reported. The genetic linkage map and QTL positions were drawn using MapChart 2.2 [50].

## 5. Conclusions

In this study, we report a genetic linkage map of *E. ulmoides* constructed by SRAP, AFLP, ISSR and SSR markers. This genetic linkage map provided an adequate coverage of the *E. ulmoides* genome for QTL analysis. A saturated genetic linkage map will be constructed by adding more co-dominant and functional markers. The QTL analysis provided a better genetic understanding for growth-related traits of *E. ulmoides* seedlings. Projects have been initiated to use the genetic linkage map to identify QTLs controlling other biological and economically important traits, and this will allow the potential of marker-assisted selection in the improvement of *E. ulmoides* cultivars.

## Figures and Tables

**Table 1.** Primer sequences used in the sequence-related amplified polymorphism analysis.

Forward primer				Reverse primer	
	
Name	Sequence	Name	Sequence	Name	Sequence
me1	ATA	me33	GAA	em1	AAT
me2	AGC	me34	GAT	em2	TGC
me3	AAT	me35	GAG	em3	GAC
me4	ACC	me36	GAC	em4	TGA
me5	AAG	me37	GTA	em5	AAC
me6	ACA	me38	GTT	em6	GCA
me7	ACG	me39	GTG	em7	CAA
me8	ACT	me40	GTC	em8	CAC
me9	AGG	me41	GGA	em9	CAG
me10	AAA	me42	GGT	em10	CAT
me11	AAC	me43	GGG	em11	CTA
me12	AGA	me44	GGC	em12	CTC
me13	ATG	me45	GCA	em13	CTG
me14	ATC	me46	GCT	em14	CTT
me15	ATT	me47	GCG	em15	GAT
me16	AGT	me48	GCC	em16	GTC
me17	TAA	me49	CAA	em17	AAG
me18	TAT	me50	CAT	em18	ATC
me19	TAG	me51	CAG	em19	AGA
me20	TAC	me52	CAC	em20	ACT
me21	TTA	me53	CTA	em21	TAC
me22	TTT	me54	CTT	em22	TTG
me23	TTG	me55	CTG	em23	TGT
me24	TTC	me56	CTC	em24	TCG
me25	TGA	me57	CGA	em25	GAA
me26	TGT	me58	CGT	em26	GTG
me27	TGG	me59	CGG	em27	GGA
me28	TGC	me60	CGC	em28	GCT
me29	TCA	me61	CCA	em29	CGA
me30	TCT	me62	CCT	em30	CGT
me31	TCG	me63	CCG	em31	CCA
me32	TCC	me64	CCC	em32	CCT

The primers consist of the core sequences and three selective nucleotides at the 3′ end. The core sequence of the forward primers is TGAGTCCAAACCGG. The core sequence of the reverse primers is GACTGCGTACGAATT. Only the three selective nucleotides are presented. A total of 2048 SRAP primer combinations were used to screen for polymorphisms.

**Table 2.** Primer sequences used in the amplified fragment length polymorphism analysis.

*EcoR*I primers		*Mse*I primers	
	
Name	Sequence	Name	Sequence
E1	AAC	M1	CAA
E2	AAG	M2	CAC
E3	ACA	M3	CAG
E4	ACT	M4	CAT
E5	ACC	M5	CTA
E6	ACG	M6	CTC
E7	AGC	M7	CTG
E8	AGG	M8	CTT

The adaptor sequences were: 5′-CTCGTAGACTGCGTACC-3′, 3′-CTGACGCATGGTTAA-5′ (*EcoR*I adaptors), 5′-GACGATGAGTCCTGAG-3′, 3′-TACTCAGGACTCAT-5′ (*Mse*I adaptors). The *EcoR*I pre-amplification primer sequence was 5′-GACTGCGTACCAATTC-3′. The *Mse*I pre-amplification primer sequence was 5′-GATGAGTCCTGAGTAA-3′. The three selective nucleotides were presented. A total of 64 AFLP primer combinations were used to screen for polymorphisms.

**Table 3.** Primer sequences used in the inter-simple sequence repeat analysis.

Name	Sequence	Annealing temperature
UBC808	C(AG)^8^C	56.0
UBC811	(GA)^8^C	43.6
UBC830	(TG)^8^G	50.0
UBC834	(AG)^8^YT	56.0
UBC835	(AG)^8^YC	43.6
UBC840	(GA)^8^YT	56.0
UBC842	(GA)^8^YG	54.7
UBC853	(TC)^8^RT	46.3
UBC860	(TG)^8^RA	56.0
UBC866	CT(CCT)^5^C	52.6
UBC867	(GGC)^6^	41.4
UBC868	(GAA)^6^	46.3
UBC873	(GACA)^4^	50.0
UBC880	(GGAGA)^3^	50.0
UBC881	(GGGGT)^3^	50.0
UBC886	VDV(CT)^7^	52.6

R = (A, G); Y = (C, T); D = (A, G, T); V = (A, C, G).

**Table 4.** Polymorphic markers detected and their segregation ratios.

Marker type a	No. of primer combinations b	No. of polymorphic markers	Lmxll (1:1) c	Nnxnp (1:1) d	Hkxhk (3:1) e	Hkxhk (1:2:1) f	Egxef (1:1:1:1) g	Abxcd (1:1:1:1) h	Distorted markers (*p* < 0.05)
SRAP	131	1604	305	326	382	18	13	8	552
AFLP	18	295	141	108	22	0	0	0	24
ISSR	16	111	27	23	31	0	0	0	30
SSR	17	132	33	42	26	6	7	1	17
Total	182	2142	506	499	461	24	20	9	623

a *SRAP* sequence-related amplified polymorphism, *AFLP* amplified fragment length polymorphism, *ISSR* inter-simple sequence repeat, *SSR* simple sequence repeat;

b *No. of primer combinations* primer combination for SRAP, AFLP and SSR, single primer for ISSR;

c *lmxll (1:1)* lmxll marker, present in the female parent only, segregating 1:1 (ll:lm) in the progeny;

d *nnxnp (1:1)* nnxnp marker, present in the male parent only, segregating 1:1 (nn:np) in the progeny;

e *hkxhk (3:1)* hkxhk marker, heterozygous in both parents, segregating 3:1 (hh+hk+h-:kk) in the progeny;

f *hkxhk (1:2:1)* hkxhk marker, heterozygous in both parents, segregating 1:2:1 (hh:hk:kk) in the progeny;

g *egxef (1:1:1:1)* egxef marker, heterozygous in both parents, segregating 1:1:1:1 (ee:ef:eg:fg) in the progeny;

h *abxcd (1:1:1:1)* abxcd marker, heterozygous in both parents, segregating 1:1:1:1 (ac:ad:bc:bd) in the progeny.

**Table 5.** Linkage group (LG), markers mapped and marker density for the genetic linkage map of DZ0901 population.

Linkage group	Length (cM)	No. of markers	SRAPs	AFLPs	ISSRs	SSRs	Mean distance (cM)
LG1	153.0	106	70	7	0	29	1.5
LG2	194.0	77	9	65	0	3	2.6
LG3	189.5	76	74	0	2	0	2.5
LG4	96.5	65	64	0	0	1	1.5
LG5	60.5	49	37	0	5	7	1.3
LG6	123.5	45	0	45	0	0	2.8
LG7	82.8	37	37	0	0	0	2.3
LG8	72.9	26	25	0	0	1	2.9
LG9	88.9	25	12	0	0	13	3.7
LG10	66.2	25	25	0	0	0	2.8
LG11	70.7	21	20	0	1	0	3.5
LG12	92.9	21	21	0	0	0	4.7
LG13	67.1	19	18	0	1	0	3.7
LG14	60.7	13	13	0	0	0	5.1
LG15	57.3	12	11	0	1	0	5.2
LG16	54.3	12	12	0	0	0	4.9
LG17	89.6	11	10	0	1	0	9.0
LG18	106.5	10	10	0	0	0	11.8
LG19	41.8	10	10	0	0	0	4.6
LG20	19.9	10	6	0	4	0	2.2
LG21	63.5	8	7	0	1	0	9.1
LG22	92.8	8	8	0	0	0	13.3
LG23	49.1	8	8	0	0	0	7.0
LG24	92.0	7	3	0	2	2	15.3
LG25	47.0	5	5	0	0	0	11.8
Total	2133.0	706	515	117	18	56	3.1

*SRAP* sequence-related amplified polymorphism; *AFLP* amplified fragment length polymorphism; *ISSR* inter-simple sequence repeat; *SSR* simple sequence repeat.

**Table 6.** Mean, standard deviation (SD), range and coefficient of variation (CV) for the growth traits.

Trait	Mean	SD	Minimum	Maximum	CV (%)
Height 2010 (cm)	39.3	16.8	9.0	85.0	42.8
Height 2011 (cm)	138.7	36.6	50.0	216.0	26.4
Height 2012 (cm)	224.8	48.4	120.0	310.0	21.5
Height 2013 (cm)	332.0	74.4	170.0	480.0	22.4
Basal diameter 2010 (mm)	4.9	1.7	1.3	9.4	34.0
Basal diameter 2011 (mm)	11.9	3.1	3.9	19.6	25.8
Basal diameter 2012 (mm)	19.2	4.6	10.0	30.4	24.0
Basal diameter 2013 (mm)	22.4	6.1	10.4	38.3	27.2

**Table 7.** Pearson correlation coefficients between the growth traits.

Traits	Height 2011	Height 2012	Height 2013	Basal diameter 2010	Basal diameter 2011	Basal diameter 2012	Basal diameter 2013
Height 2010	0.52 *	−0.04	−0.02	0.80 *	0.51 *	0.02	0.03
Height 2011		0.04	0.02	0.51 *	0.83 *	0.03	0.15
Height 2012			0.70 *	−0.02	0.03	0.66 *	0.75 *
Height 2013				0.05	−0.04	0.64 *	0.72 *
Basal diameter 2010					0.47 *	0.01	0.04
Basal diameter 2011						0.10	0.10
Basal diameter 2012							0.92 *

* *p* < 0.01.

**Table 8.** QTLs of growth traits detected in the DZ0901 population.

QTL a	Linkage group	Peak position (cM) b	LOD peak c	Marker d	% Var.expl. e	KW f
Height 2010						

*Dht0-1*	LG18	106.5	7.8 **	em11me14–300c	17.1	****

Height 2011						

*Dht1-1*	LG10	14.9	4.4 **	em39me7–750	29.7	-
*Dht1-2*	LG10	27.1	4.4 **	em39me7–330	27.7	*
*Dht1-3*	LG12	40.1	3.1	em9me7–230	22.8	-

Height 2012						

*Dht2-1*	LG9	62.0	3.2	DZ126–280	12.6	-
*Dht2-2*	LG13	14.3	3.1	em13me4–360	12.4	****
*Dht2-3*	LG22	14.0	3.3	em15me23–360	33.3	-

Height 2013						

*Dht3-1*	LG9	62.0	3.9	DZ126–280	13.5	-
*Dht3-2*	LG21	57.4	3.3	UBC881–820	26.6	-
*Dht3-3*	LG22	0.0	3.8	em6me8–260	25.3	-
*Dht3-4*	LG24	0.0	4.3	DZ200–350	27.1	-

Basal diameter 2010						

*Dbd0-1*	LG18	72.1	3.8	em3me14–700	29.8	**
*Dbd0-2*	LG18	106.5	4.7 **	em11me14–300c	13.4	****

Basal diameter 2011						

*Dbd1-1*	LG12	40.1	3.0	em9me7–230	20.2	-

Basal diameter 2012						

*Dbd2-1*	LG1	153.0	3.0	em12me11–300	17.7	-
*Dbd2-2*	LG21	58.4	3.2	em5me7–530	25.1	**
*Dbd2-3*	LG22	0.0	3.6	em6me8–260	21.4	**

Basal diameter 2013						

*Dbd3-1*	LG1	153.0	3.0	em12me11–300	16.8	-

a QTL named using an abbreviation of the trait (*Dht* Height, *Dbd* Basal diameter), followed by the year (0 for 2010, 1 for 2011, 2 for 2012, 3 for 2013) and the QTL number;

b Peak position log of odds (LOD) peak position;

c LOD peak maximum LOD value;

** LOD value significant at *p* < 0.05 based on 1000 genome-wide permutation tests;

d Marker marker name nearest to the QTL position;

e % Var. expl. proportion of the total phenotypic variance explained by the QTL;

f KW Kruskal-Wallis significance level, given by the *p* values (* 0.1; ** 0.05; **** 0.0005).
